# Peripheral and central regulation of neuro–immune crosstalk

**DOI:** 10.1186/s41232-024-00352-3

**Published:** 2024-09-26

**Authors:** Mayuko Izumi, Yoshimitsu Nakanishi, Sujin Kang, Atsushi Kumanogoh

**Affiliations:** 1 Department of Respiratory Medicine and Clinical Immunology, Graduate School of Medicine, Osaka University, Osaka, 565-0871 Japan; 2 Department of Immunopathology, World Premier International Research Center Initiative Immunology Frontier Research Center (WPI-IFReC), Osaka University, Osaka, 565-0871 Japan; 3 Department of Advanced Clinical and Translational Immunology, Graduate School of Medicine, Osaka University, Osaka, 565-0871 Japan; 4 Integrated Frontier Research for Medical Science Division, Institute for Open and Transdisciplinary Research Initiatives (OTRI), Osaka University, Osaka, 565-0871 Japan; 5 Laboratory of Immune Regulation, WPI-IFReC, Osaka University, Osaka, 565-0871 Japan; 6 Center for Infectious Diseases for Education and Research (CiDER), Osaka University, Osaka, 565-0871 Japan; 7 Japan Agency for Medical Research and Development – Core Research for Evolutional Science and Technology (AMED–CREST), Osaka University, Osaka, 565-0871 Japan; 8 Center for Advanced Modalities and DDS (CAMaD), Osaka University, Osaka, 565-0871 Japan

**Keywords:** Neuro–immune crosstalk, Peripheral nervous system, Central nervous system, Brain, Skin, Lung, Adipose tissue, Intestine

## Abstract

The neural and immune systems sense and respond to external stimuli to maintain tissue homeostasis. These systems do not function independently but rather interact with each other to effectively exert biological actions and prevent disease pathogenesis, such as metabolic, inflammatory, and infectious disorders. Mutual communication between these systems is also affected by tissue niche-specific signals that reflect the tissue environment. However, the regulatory mechanisms underlying these interactions are not completely understood. In addition to the peripheral regulation of neuro–immune crosstalk, recent studies have reported that the central nervous system plays essential roles in the regulation of systemic neuro–immune interactions. In this review, we provide an overview of the molecular basis of peripheral and systemic neuro–immune crosstalk and explore how these multilayered interactions are maintained.

## Background

Both neural and immune systems are indispensable for the physiological functions of various tissues. Recent studies have revealed that these systems function in a coordinated manner, establishing neuro–immune crosstalk. Disruption of this crosstalk leads to various diseases, including infectious, autoimmune, and metabolic disorders. The nervous and immune systems share signaling pathways mediated by neurotransmitters, neuropeptides, and cytokines, which facilitate rapid peripheral responses to external stimuli. In addition to neuro–immune crosstalk in peripheral tissues, a growing body of literature highlights that the central nervous system (CNS) also regulates peripheral immune responses. For instance, information regarding peripheral inflammation is encoded in specific neural circuits within the brain [1]. Moreover, signals from the brain, transmitted via the autonomic nervous system (ANS), regulate not only immediate immune responses but also peripheral immune niche formation [2]. Furthermore, our recent findings indicate that semaphorin 6D (SEMA6D), an axon-guidance molecule, is essential for the maintenance of synaptic maturation and γ-aminobutyric acid (GABA) signaling in the amygdala, thereby regulating hematopoietic and inflammatory responses via sympathetic outputs [3]. Therefore, the CNS, including the brain, establishes systemic neuro–immune crosstalk through bidirectional signaling in peripheral tissues. This review summarizes recent advances in our understanding of both peripheral and central neuro–immune crosstalk.

## Neuro–immune crosstalk in the skin

**Fig. 1:**
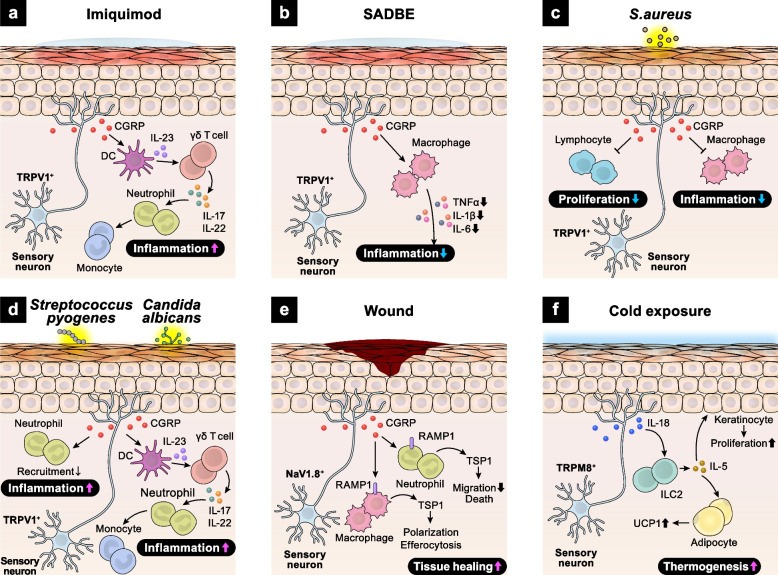
Neuro–immune crosstalk in the skin. **a** Imiquimod induces skin inflammation by enhancing CGRP secretion from TRPV1+ sensory neurons. CGRP promotes dendritic cell-derived IL-23 production and then activates γδ T cells to produce IL-17 and IL-22. **b** SADBE activates TRPV1+ sensory neurons to release CGRP, which in turn inhibits the secretion of inflammatory cytokines from dermal macrophages. **c** S.aureus stimulates TRPV1+ sensory neurons to secrete CGRP, leading to suppression of macrophage inflammation and lymphocyte proliferation. **d** Streptococcus pyogenes increases TRPV1+ sensory neuron-derived CGRP release, which inhibits neutrophil recruitment. When infected with Candida albicans, TRPV1+ sensory neurons release CGRP. CGRP promotes the production of IL-23 from dermal dendritic cells, which then activates γδ T cells and exacerbates skin inflammation. **e** Skin injury drives the release of NAV1.8+ sensory neuron-derived CGRP. CGRP binds to RAMP1 expressed on macrophages and neutrophils, promoting tissue healing. **f** Cold exposure induces IL-18 production from TRPM8+ sensory neurons. IL-18 activates skin ILC2s to secrete IL-5. ILC2-derived IL-5 promotes proliferation of keratinocytes and UCP1 expression in hypodermal adipocytes, resulting in enhanced thermogenesis

The skin functions not only as a barrier but also as a sensory organ, being highly innervated by sensory nerves. Innate immune cells, such as macrophages, neutrophils, dendritic cells, and mast cells, reside in the epidermis and interact with sensory nerves to maintain skin barrier integrity. Transient receptor potential vanilloid 1 (TRPV1)-expressing sensory neurons release calcitonin gene-related peptide (CGRP), which activates adjacent dendritic cells to elicit inflammatory responses [4]. Imiquimod, a toll-like receptor 7 (TLR7) agonist, induces skin inflammation resembling human psoriasis via activation of the interleukin 23 (IL-23)/interleukin 17 (IL-17) axis [4, 5]. The IL-23/IL-17 axis is one of the major inflammatory pathways and plays key roles in establishing chronic skin inflammation [6]. Upon imiquimod exposure, CGRP released from TRPV1 + sensory neurons induces IL-23 production in dendritic cells, which subsequently enhances IL-17 release from dermal γδ T cells, exacerbating skin inflammation [4, 5] (Fig. 1a). Additionally, in a squaric acid dibutylester (SADBE)-induced contact dermatitis model, sensory neurons directly detect SADBE via TRPV1 and suppress inflammatory cytokine production by dermal macrophages [7] (Fig. 1b).

TRPV1^+^ sensory neurons also respond to bacterial and fungal components, regulating immune responses via CGRP signaling [7]. *Staphylococcus aureus* (*S.aureus*)-derived N-formylated peptides induce calcium flux and action potentials in TRPV1 + sensory neurons via G-protein coupled formyl peptide receptors. In addition, *S.aureus*-derived α-hemolysin promotes pore formation on the transmembrane of TRPV1 + sensory neurons, leading to ionic influx. This synergistic activation of TRPV1 + sensory neurons reduces skin inflammation by suppressing both macrophage inflammation and lymphocyte proliferation [8] (Fig. 1c). Conversely, during *Streptococcus pyogenes* infection, CGRP from TRPV1^+^ sensory neurons suppresses neutrophil recruitment and anti-microbial activity, exacerbating the infection [9] (Fig. 1d). In fungal infections, TRPV1^+^ sensory neurons detect *Candida albicans*-derived β-glucan and release CGRP, which activates the IL-23–IL-17 inflammatory axis between dendritic cells and γδ T cells [10] (Fig. 1d).

Recent studies have revealed that NaV1.8^+^ sensory neurons contribute to wound healing by suppressing inflammatory responses. Upon skin injury, CGRP from NaV1.8^+^ sensory neurons binds to the receptor activity-modifying protein 1 (RAMP1) expressed on neutrophils, monocytes, and macrophages, inhibiting their recruitment, accelerating their death, enhancing efferocytosis, and polarizing macrophages towards a pro-repair phenotype [11] (Fig. 1e).

In addition to peptidergic sensory neurons, non-peptidergic sensory neurons maintain cutaneous immune homeostasis [12]. Mas-related G protein-coupled receptor D (MrgprD)-expressing non-peptidergic neurons release glutamate, which binds to glutamate receptors on mast cells, suppressing mast cell degranulation. Langerhans cells (LCs), epidermal-resident antigen-presenting cells, support the survival of MrgprD^+^ neurons. Ablation of LCs or MrgprD^+^ neurons exacerbates *S. aureus* infection and contact hypersensitivity due to enhanced mast cell activation, indicating the critical role of LC–non-peptidergic neuron–mast cell interaction in maintaining cutaneous integrity in diverse disease contexts.

Intact skin is crucial for maintaining thermal homeostasis. A recent study revealed a unique function of cutaneous group 2 innate lymphoid cells (ILC2s) in sensory neuron crosstalk during thermogenesis [13] (Fig. 1f). Cold stimuli activate cutaneous sensory neurons expressing transient receptor potential cation channel subfamily M member 8 (TRPM8), a cold-sensing receptor, inducing interleukin-18 (IL-18) production. Neuronal IL-18 activates skin ILC2s to promote interleukin-5 (IL-5) secretion, which induces uncoupling protein 1 (UCP1) expression in hypodermal adipocytes, enhancing thermogenesis. Taken together, the intricate crosstalk between sensory neurons and leukocytes plays key roles in maintaining skin homeostasis in mice.

## Neuro–immune crosstalk in the lung

**Fig. 2:**
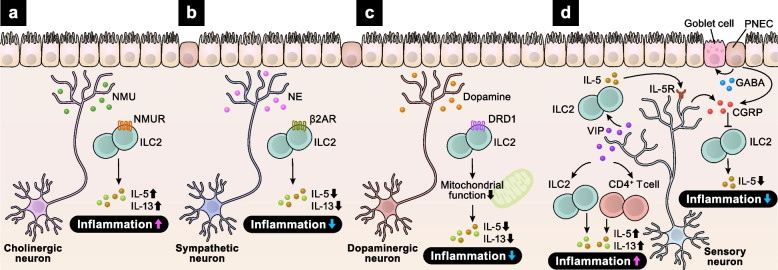
Neuro–immune crosstalk in the lung. **a**–**d** Upon allergen exposure, various types of neurons activate immune responses in the lung.Cholinergic neuron-derived NMU induces IL-5 and IL-13 secretion from ILC2s via NMUR, exaggerating lung inflammation (**a**). NE from sympathetic neurons and dopamine from dopaminergic neurons inhibit ILC2 responses via β2AR (**b**) and DRD1 (**c**), respectively. Sensory neuron-derived VIP activates ILC2s and CD4^+^ T cells to produce IL-5 and IL-13, which exacerbates allergic inflammation. In turn, ILC2-derived IL-5 binds to IL-5R on sensory neurons and promotes CGRP secretion. CGRP, derived from both sensory neurons and PNECs, inhibits ILC2 responses and suppresses airway inflammation (**d**)

The lungs, highly innervated and densely populated with immune cells, are constantly exposed to environmental cues. Neuro–immune crosstalk plays crucial roles in asthma development. ILC2s secrete type 2 cytokines, including IL-5 and interleukin 13 (IL-13), inducing asthmatic responses, such as smooth muscle contraction, eosinophil infiltration, and goblet cell hyperplasia [14, 15]. Recent studies have revealed that ILC2 functions are tightly regulated by neurotransmitters and neuropeptides such as neuromedin U (NMU), norepinephrine (NE), vasoactive intestinal peptide (VIP), and CGRP. ILC2s colocalize with NMU-expressing cholinergic neurons in the gut and lungs and uniquely express the NMU receptor 1 (NMUR1) (Fig. 2a). NMU-mediated ILC2 activation induces immediate type 2 responses [16–18]. ILC2s also express both nicotinic ACh receptors (nAChRs) and muscarinic ACh receptors (mAChRs), resulting in complex functions of ACh in the regulation of ILC2 responses. Although ACh signaling via α7-nAChR suppresses ILC2-mediated inflammation, ACh signaling via other ACh receptors enhances inflammatory responses of ILC2s [19–22]. Additionally, ILC2s express β2-adrenergic receptor (β2AR) and colocalize with sympathetic neurons [23]. Unlike NMU signaling, NE signaling via β2AR suppresses ILC2-mediated allergic airway inflammation (Fig. 2b). Dopamine–DRD1 signaling also impairs ILC2 responses by suppressing mitochondrial oxidative phosphorylation [24] (Fig. 2c). Conditional deletion of DRD1 in ILC2s exacerbates IL-33–induced airway inflammation in mice. Conversely, intranasal administration of dopamine ameliorates airway inflammation by suppressing ILC2 responses.

Lung nociceptors and ILC2s form a vicious cycle of allergic inflammation [25]. VIP activates ILC2s and CD4^+^ T cells via the vasoactive intestinal peptide receptor type 2 (VPAC2), promoting allergic airway inflammation in mice (Fig. 2d). IL-5 from these cells enhances VIP release by nociceptors, thereby perpetuating allergic inflammation. CGRP also plays key roles in regulating ILC2 function. Sensory neurons and pulmonary neuroendocrine cells (PNECs) mainly produce CGRP in the lungs, while a subset of ILC2s express CGRP and its receptor CALCRL/RAMP1. CGRP potently suppresses alarmin-driven type 2 cytokine production and proliferation by ILC2s [26, 27]. Recent research revealed that sensory neuron-intrinsic Janus kinase 1 signaling promotes CGRP secretion, thereby suppressing ILC2-mediated allergic lung inflammation [28]. However, CGRP can enhance mucosal type 2 responses, including ILC2 activation, in a mouse model of allergic asthma [29]. These opposing effects of CGRP on ILC2 function may depend on the cellular source or experimental context.

PNECs are specialized components of the lung epithelium that form neuroepithelial bodies (NEBs). PNECs receive sensory and parasympathetic innervations, thereby producing hormones and neuropeptides upon neurological and chemical stimuli [30]. At airway branch points, ILC2s interact with nearby PNECs [29]. PNECs secrete CGRP and GABA, which activate ILC2s and induce goblet cell hyperplasia. Mice lacking PNECs exhibit attenuated allergic responses. In accordance, PNECs are increased in the lungs of human patients with asthma. Collectively, altered neuro–immune interactions are the key components of allergic disease pathogenesis in the lung.

## Neuro–immune crosstalk in the adipose tissue

**Fig. 3:**
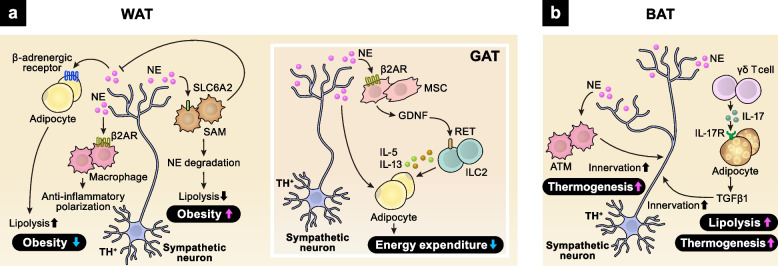
Neuro–immune crosstalk in the adipose tissue. **a** In WAT, TH^+^ sympathetic neuron-derived NE binds to β-adrenergic receptor expressed on adipocytes and ATMs, promoting lipolysis and macrophage polarization to limit the development of obesity. On the other hand, SAM, a unique subset of adipose macrophages, promotes obesity through degrading NE. NE also induces GDNF secretion from MSCs in GAT. GDNF promotes IL-5 and IL-13 production from ILC2s through binding to RET. **b **In BAT, TH^+^ sympathetic neuron-derived NE activates ATMs to promote sympathetic innervation, enhancing thermogenesis. In addition, γδ T cell–adipocyte interaction increases sympathetic innervation via TGFβ1 signaling

The adipose tissue contains a bunch of immune cells, such as macrophages, ILC2s, and γδ T cells. Immune responses mediated by these cells are essential for the metabolic and endocrine functions of the adipose tissue. In addition, the adipose tissue is highly innervated and regulated by the ANS. Especially, in collaboration with immune cells, sympathetic nerves play crucial roles in maintaining metabolic homeostasis in the adipose tissue. Sympathetic signals act on both adipocytes and immune cells to suppress the development of metabolic disorders. Catecholamines bind to β-adrenergic receptors on adipocytes, activating the protein kinase A (PKA)–cyclic adenosine monophosphate (cAMP) cascade, which enhances lipolysis [31] (Fig. 3a). Pro-inflammatory polarization of adipose tissue macrophages (ATMs) is a hallmark of obesity. The catecholamine–β2AR–PKA–cAMP axis promotes the anti-inflammatory polarization of ATMs, suppressing local inflammation and obesity [32, 33]. In addition, recent studies revealed unique functions of ATMs in NE metabolism [34, 35]. A subset of ATMs, sympathetic neuron-associated macrophages (SAMs), mediate the clearance of NE and promote the pathogenesis of obesity in mice and humans.

White adipose tissue (WAT) is a specialized organ for the storage of fatty acids as an energy source, whereas brown adipose tissue (BAT) uses fatty acids for ATP synthesis and thermogenesis. Sympathetic nerves densely innervate BAT and tightly regulate its function. Recent studies have shown that adipose immune cells play key roles in controlling sympathetic innervation in BAT [36, 37] (Fig. 3b). Firstly, ATMs support sympathetic innervation in BAT [36]. Mice lacking the nuclear transcription regulator Mecp2 in ATMs display spontaneous obesity due to diminished sympathetic innervation and impaired thermogenesis in BAT. Mechanistically, the loss of *Mecp2* enhances the expression of Plexin-A4 in BAT-resident macrophages, promoting the axonal repulsion of sympathetic neurons expressing semaphorin 6A. Secondly, γδ T cells, in collaboration with parenchymal cells, also promote sympathetic innervation in several tissues, including BAT [37]. γδ T cell-derived IL-17F binds to IL-17 receptor C (IL-17RC) highly expressed on parenchymal cells and drives the expression of TGFβ1, promoting sympathetic innervation. Adipose-specific loss of IL-17RC signaling diminishes sympathetic innervation in BAT, impairs BAT thermogenesis, and aggravates High-fat diet (HFD)-induced obesity, indicating the critical involvement of γδ T cell-mediated neuronal regulation in metabolic disorders.

Sympathetic neurons also act on adipose mesenchymal cells to maintain the immune niche in the gonadal adipose tissue (GAT) [38] (Fig. 3a). Sympathetic signals induce the expression of glial-derived neurotrophic factor (GDNF) in adipose mesenchymal cells (MSCs). In turn, mesenchyme-derived GDNF activates adipose ILC2s that express the tyrosine kinase receptor RET, a functional receptor for GDNF, thereby shaping energy expenditure and metabolic homeostasis.

### Neuro–immune crosstalk in the intestine

**Fig. 4:**
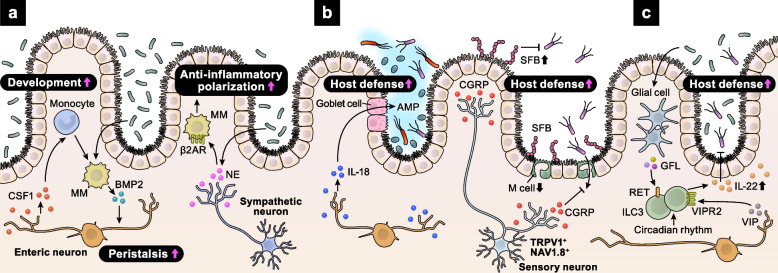
Neuro–immune crosstalk in the intestine. **a** The microbiota increases the levels of MM-derived BMP2, which acts on enteric neurons to control peristalsis. BMP2 also enhances CSF1 secretion from enteritic neurons, supporting the development of MMs. In addition, the microbiota supports anti-inflammatory polarization of MMs via sympathetic activation. **b** Upon *Salmonella* infection, enteric neuron-derived IL-18 increases AMP secretion from goblet cells, supporting host defense. Sensory neuron-derived CGRP also enhances host defense against *Salmonella *by increasing the levels of SFB and decreasing the density of M cells. **c** Enteric glial cells sense the microbiota and secrete GFLs, which increase ILC3-derived IL-22 via RET, suppressing intestinal inflammation. Circadian rhythmicity maintains the levels of ILC3-derived IL-22. In addition to ILC3-intrinsic circadian regulation, cyclic patterns of food intake affect IL-22 secretion from ILC3s via VIP–VIPR2 signaling axis

The intestine is densely innervated by the ANS, which includes the parasympathetic, sympathetic, and enteric nervous systems. Constantly exposed to microbial stimuli, neurons regulate intestinal homeostasis in cooperation with immune cells. Intestinal macrophages and enteric neurons interact to control peristalsis [39] (Fig. 4a). In the intestinal muscularis, macrophages secrete bone morphogenetic protein 2 (BMP2), which activates BMP receptors on enteric neurons, altering smooth muscle contractions and colon peristaltic activity. In turn, enteric neurons secrete colony stimulatory factor 1 (CSF1), promoting macrophage development. The reciprocal relationship between muscularis macrophages (MMs) and enteric neurons is maintained by microbial commensals. MMs also receive sympathetic signals via β2AR [40] (Fig. 4a). Luminal bacterial infection results in the activation of extrinsic sympathetic neurons in the gut muscularis, promoting NE release. NE acts on β2AR^+^ MMs, driving their polarization to a tissue-protective phenotype. Furthermore, polarized anti-inflammatory MMs protect enteric neurons from caspase-11-dependent death during bacterial infections [41]. Thus, microbial-driven integration of neural and immune responses is crucial for maintaining intestinal function and host defense.

MMs also contribute to the development of the enteric nervous system (ENS) in mice and humans [42]. They engulf synapses and enteric neurons during early postnatal development, refining the ENS. In adulthood, MMs receive ENS-derived TGFβ signaling, acquiring a neuro-supportive phenotype. Thus, the ENS–MM interaction has developmental stage-dependent functions.

Importantly, enteric neurons themselves participate in host defense [43] (Fig. 4b). IL-18 from enteric neurons, but not from immune or epithelial cells, is essential for homeostatic goblet cell antimicrobial peptide (AMP) production. Loss of neural IL-18 secretion impairs the host defense against invasive *Salmonella* infection. In addition to autonomic neurons, gut-innervating nociceptor sensory neurons also contribute to sensing and defense against enteric pathogens by interacting with epithelial cells and intestinal microbiota [44] (Fig. 4b). Dorsal root ganglia nociceptors respond directly to *Salmonella* infection by secreting CGRP. CGRP decreases the density of microfold (M) cells, specialized epithelial cells in the ileum Peyer’s patch (PP) follicle-associated epithelia (FAE), limiting *Salmonella* entry. Moreover, CGRP maintains levels of segmented filamentous bacteria (SFB), intestine-resident microbes that mediate resistance to *Salmonella* colonization and invasion.

Group 3 innate lymphoid cells (ILC3s) produce pro-inflammatory cytokines, including IL-17A, interleukin 22 (IL-22), and tumor necrosis factor, regulating mucosal homeostasis and anti-microbial defense [45]. Recent studies have revealed the neural regulation of ILC3 functions. Enteric ILC3s express the neuroregulatory tyrosine kinase receptor RET and are adjacent to enteric glial cells expressing neurotrophic factors, such as glial-derived neurotrophic factor family ligands (GFLs) [46] (Fig. 4c). Enteric glial cells detect and respond to microbial cues in a myeloid differentiation primary response 88 (MYD88)-dependent manner by secreting GFLs. GFLs promote the production of ILC3-derived IL-22, which suppresses intestinal inflammation and infection. In addition to enteric glial cells, the vagal system regulates ILC3 functions in mice and humans [47]. In *Escherichia coli*-infected mice, vagotomy decreases peritoneal ILC3s and increases inflammatory peritoneal lipid mediators, delaying inflammation resolution. Mechanistically, acetylcholine upregulates the biosynthesis of pro-resolving protectins in ILC3s.

Besides the peripheral neural regulation of ILC3s, brain-mediated circadian signaling controls ILC3 homeostasis [48]. ILC3s highly express circadian genes and exhibit a diurnal rhythm in response to light signals, necessary for their effector functions and maintenance of epithelial integrity and microbiome [48–51]. The circadian rhythm of ILC3s is disrupted in patients with inflammatory bowel disease (IBD), highlighting the importance of ILC3-intrinsic circadian regulation in suppressing spontaneous inflammation [49]. Disruption of brain rhythmicity, whether surgically or genetically induced, disrupts ILC3 circadian oscillations [48], indicating that the brain translates environmental light signals into enteric ILC3 responses. Cyclic patterns of food intake also affect ILC3 functions [51]. Upon food intake, enteric neurons secrete VIP, which binds to its receptor VIPR2, highly expressed in ILC3s. The VIP–VIPR2 signaling axis enhances IL-22 production from ILC3s and strengthens the barrier function of the epithelium. Together, neuro–immune interactions are essential for detecting microbial environment and maintaining physiological homeostasis in the intestine.

### Systemic neuro–immune crosstalk

**Fig. 5:**
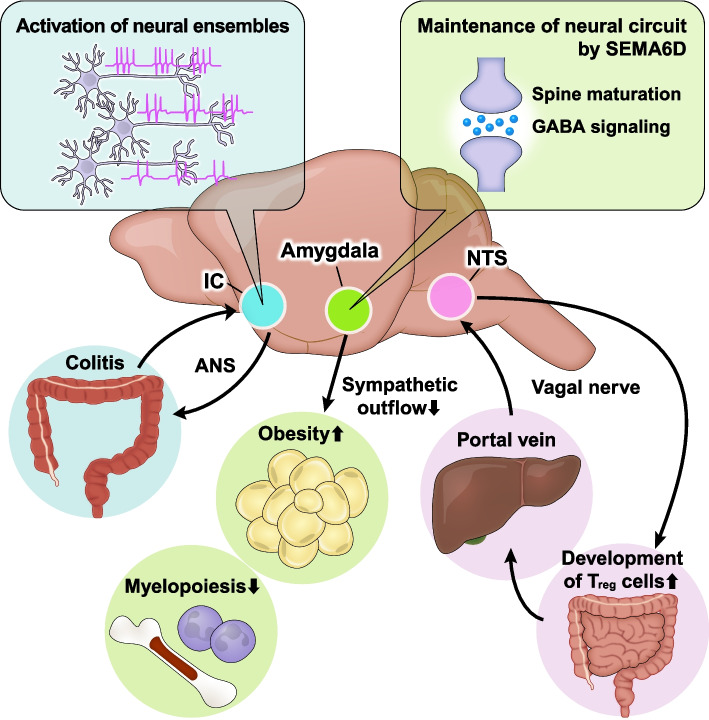
Systemic neuro–immune crosstalk Several brain regions coordinate neuro–immune interactions in peripheral tissues. Neural circuits in IC record and retrieve peripheral inflammation, such as colitis and peritonitis. The maintenance of amygdalar synaptic homeostasis is essential for orchestrating systemic metabolism and hematopoiesis. NTS communicates with the intestine and the liver via vagal nerves, thereby establishing the intestinal immune niche

In addition to the local neuro–immune crosstalk in the peripheral tissues, recent studies have highlighted the brain’s essential roles in regulating peripheral neuro–immune interactions (Fig. 5). The vago-vagal liver–brain–gut reflex arc is crucial for the differentiation and maintenance of regulatory *T* (*T*_reg_) cells in the gut [52]. The liver continuously senses the gut microenvironments via the portal circulation. Metabolites, bacterial products, and nutrients activate the hepatic sensory afferents of the vagus nerve via the mechanistic target of rapamycin complex 1. This sensory information from the gut microenvironment is transmitted to the nucleus tractus solitarius (NTS) of the brainstem [53]. Vagal parasympathetic nerves and enteric neurons activate antigen-presenting cells (APCs) through mAChR, enhancing aldehyde dehydrogenase expression and retinoic acid synthesis in mice and humans [52]. APC-derived retinoic acid supports the development of *T*_reg_ cells in the gut [54]. Surgical ablation of the left vagal sensory afferents from the liver to the brainstem reduces the population of *T*_reg_ cells in the gut and increases susceptibility to colitis in mice, indicating that the liver–brain–gut neural reflex maintains the immune niche in the gut, thereby suppressing inflammatory diseases.

The brain also records and recalls peripheral immune responses [1]. Dextran sulfate sodium (DSS)-induced colitis and zymosan-induced peritonitis activate distinct neuronal ensembles in the insular cortex (IC), storing information on peripheral inflammation as neuronal activity. These neuronal ensembles project to the dorsal motor nucleus of the vagus and rostral ventrolateral medulla, both of which control the ANS. Chemogenetic reactivation of these IC neurons recapitulates the inflammatory state in the colon via the ANS. Moreover, chemogenetic inhibition of IC activity attenuates DSS-induced colitis, implying the therapeutic potential of IC-targeted manipulation in treating colitis, including IBD.

Another recent study uncovered a specific body–brain neuronal circuit that senses and alleviates systemic inflammation [2]. Intraperitoneal injection of lipopolysaccharide (LPS) increases neuronal activity in the caudal NTS (cNTS) of the brainstem via vagal activation by pro-inflammatory and anti-inflammatory cytokines. In turn, cNTS neurons modulate immunity and suppress systemic inflammation. Activation or inhibition of this body–brain circuit results in suppressed or enhanced systemic inflammation, respectively.

Recently, we reported the essential roles of the amygdala in coupling emotional, metabolic, and inflammatory responses under stress conditions [3]. The amygdala orchestrates emotional, physiological, and behavioral responses in the presence of imminent threats [55]. Upon emotional stimuli, the amygdala activates the sympathetic nervous system, promoting the myeloid skewing of immune cells and the development of atherosclerosis in humans [56, 57]. We identified semaphorin 6D (SEMA6D) as a critical regulator of amygdalar neural integrity [3]. SEMA6D is highly expressed in the central amygdala (CeA), whereas Plexin-A4, one of the receptors for SEMA6D, is selectively expressed in the basolateral amygdala (BLA). The SEMA6D–Plexin-A4 signaling axis mediates synaptic maturation and GABA transmission in the amygdala. Loss of amygdalar SEMA6D increases immature spines in BLA and decreases the levels of GABA in CeA, resulting in enhanced anxiogenic responses. In addition, under HFD feeding, *Sema6d* ^*−/−*^ mice exhibit attenuated obesity and enhanced myelopoiesis due to elevated sympathetic tone. Taken together, these studies underscore the essential roles of each brain region in regulating the peripheral immune environment.

### Conclusions

Peripheral neuro–immune crosstalk is crucial for the development of both the nervous and immune systems, as well as for maintaining physiological organ function, host defense, and disease prevention. In addition, the brain is deeply involved in the establishment of peripheral neuro–immune crosstalk. However, our current understanding of the communication between the brain and peripheral tissues is still limited. To achieve a comprehensive understanding of systemic neuro–immune crosstalk, it is essential to determine how each brain region connects to peripheral tissues and senses peripheral environments. It also remains to be elucidated how peripheral and central neuronal information is integrated into immune cells. Moreover, most studies on brain-mediated neuro–immune crosstalk have been conducted in mice, primarily due to the challenges associated with analyzing human brain circuits. Addressing these gaps will provide new insights into the treatment of various diseases, including metabolic, infectious, and inflammatory disorders.

## Data Availability

Not applicable.

## Abbreviations

CNS: Central nervous system
ANS: Autonomic nervous system
SEMA6D: Semaphorin 6D
GABA: γ-Aminobutyric acid
TRPV1: Transient receptor potential vanilloid 1
CGRP: Calcitonin gene-related peptide
TLR7: Toll-like receptor 7
IL-23: Interleukin 23
IL-17: Interleukin 17
SADBE: Squaric acid dibutylester
*S. aureus*: *Staphylococcus aureu* *s*
RAMP1: Receptor activity-modifying protein 1
MrgprD: Mas-related G-protein-coupled receptor D
LC: Langerhans cell
ILC2: Group 2 innate lymphoid cell
TRPM8: Transient receptor potential cation channel subfamily M member 8
IL-18: Interleukin 18
IL-5: Interleukin 5
UCP1: Uncoupling protein 1
IL-13: Interleukin 13
NMU: Neuromedin U
NE: Norepinephrine
VIP: Vasoactive intestinal peptide
NMUR1: NMU receptor 1
nAChRs: Nicotinic ACh receptors.
mAChRs: Muscarinic ACh receptors.
β2AR: β2-Adrenergic receptor
VPAC2: Vasoactive intestinal peptide receptor type 2
PNEC: Pulmonary neuroendocrine cell
NEBs: Neuroepithelial bodies
PKA: Protein kinase A
cAMP: Cyclic adenosine monophosphate
ATM: Adipose tissue macrophage
TH: Thyrosine hydroxylase
SAM: Sympathetic neuron-associated macrophage
WAT: White adipose tissue
BAT: Brown adipose tissue
IL-17RC: IL-17 receptor C
HFD: High-fat diet
GAT: Gonadal adipose tissue
GDNF: Glial-derived neurotrophic factor
MSCs: Mesenchymal cells
BMP2: Bone morphogenetic protein 2
CSF1: Colony stimulatory factor 1
MM: Muscularis macrophage
ENS: Enteric nervous system
AMP: Antimicrobial peptides
M cell: Microfold cell
PP: Peyer’s patch
FAE: Follicle-associated epithelia
SFB: Segmentous filamentous bacteria
ILC3: Group 3 innate lymphoid cell
IL-22: Interleukin 22
GFL: Glial-derived neurotrophic factor family ligand
MYD88: Myeloid differentiation primary response 88
IBD: Inflammatory bowel disease
T_reg_ cell: Regulatory T cell
NTS: Nucleus tractus solitarius
APC: Antigen-presenting cell
DSS: Dextran sulfate sodium
IC: Insular cortex
LPS: Lipopolysaccharide
cNTS: Caudal nucleus tractus solitarius
CeA: Central amygdala
BLA: Basolateral amygdala
SLC6A2: Solute carrier family 6 member 2
